# Spatial characteristics of health outcomes and geographical detection of its influencing factors in Beijing

**DOI:** 10.3389/fpubh.2024.1424801

**Published:** 2024-10-16

**Authors:** Jiu Cheng, Yueying Cui, Xi Wang, Yifei Wang, Ruihua Feng

**Affiliations:** Institute of Medical Information / Medical Library, Chinese Academy of Medical Sciences and Peking Union Medical College, Beijing, China

**Keywords:** social determinants of health, health outcomes, Beijing, geographical detector, spatial characteristics

## Abstract

**Background and objective:**

Social determinants of health (SDOH) broadly influence health levels. Research on health and its influencing factors can help improve health status. There is limited research on the spatial stratified heterogeneity of health status and the interactions between the factors influencing it. This study aimed to analyze the spatial characteristics of health outcomes in Beijing and identify its influencing factors.

**Methods:**

Based on the *Healthy Beijing Initiative (2020–2030)*, we constructed health outcomes and five dimensions of the SDOH evaluation system. Our study measured the health outcomes and SDOH based on the latest data from 16 districts in Beijing in 2020–2022. We explored the spatial characteristics of health outcomes through descriptive and spatial autocorrelation analyses. Moreover, the Geographical Detector (GeoDetector) technique has been used to reveal the effect of SDOH and its interactions on health outcomes.

**Results:**

A significant spatial stratified heterogeneity of health outcomes was observed, with the health outcomes mainly exhibiting two clustering types (high–high and low–low) with positive autocorrelation. The results of the geodetector showed that social and economic factors (*q* = 0.85), healthy lifestyle (*q* = 0.68) and health service (*q* = 0.53) could mainly explain the heterogeneity of health outcomes. Social and economic factors, healthy lifestyle and healthy environment gradually became the main influential factor in health outcomes over time. Furthermore, the interaction of any two factors on health outcomes was found to be more pronounced than the impact of a single factor.

**Conclusion:**

There existed obvious spatial stratified heterogeneity of health outcomes in Beijing, which could be primarily explained by social and economic factors, and healthy lifestyle and health service.

## 1 Introduction

Life expectancy, infant mortality, mortality of children under 5 years old, and maternal mortality are important indicators for the measurement of population health: together, they reflect the health status of the population as well as the health conditions and socio-economic development of a country or region, which are vital for both policy development and health improvement programs (1). Beijing has adhered to the health-first development policy, implemented the *Healthy Beijing Initiative (2020–2030)* in 2020, aimed at comprehensively managing the factors influencing public health. Residents’ health status has continued to improve, with key health indicators now reaching the same levels as those observed in high-income countries. However, there are still spatial differences in the health status of residents of Beijing—e.g., taking life expectancy as an example, the maximum difference between districts was 3.92 years in 2022, indicating that health equity must be addressed and improved. Accordingly, exploring the spatial characteristics of health status and its influencing factors to address the regional inequalities in health status in Beijing is an important task in promoting the construction of a healthy Beijing.

There have been many studies concerning the various factors that influence public health. WHO first described the relationship between social factors and health in 1948. In 1991, Dahlgren and Whitehead developed an ecological model of social determinants of health (SDOH) (2), which includes genetic factors, personal lifestyle, and social and environmental factors. SDOH embodies the consequences of diverse social processes and norms that shape living conditions and produce a broad range of health disparities (3, 4). Traditionally, public health research has used the SDOH framework to identify important factors affecting health; among these, socioeconomic and environmental factors are the most commonly considered primary determinants (5–8). Health status exhibits a spatial pattern, and SDOH not only directly affects local health status but also indirectly affects the health status of the adjacent area from the spatial perspective. These spatial autocorrelation and lagged characteristics may violate the assumption of independence of the linear model. Although some studies have considered the spatial information contained in the data and global and local spatial regression models have been used to analyze the relationship between health indicators and explanatory covariates, the spatial stratification heterogeneity of health status has not been fully explored (9–16). Although a limited number of studies has explored the spatial stratification heterogeneity of health indicators and their drivers using geographic detectors (geodetectors), they have primarily focused on a small number of SDOH affecting individual health indicators (17, 18). Moreover, few studies have assessed health outcomes and their interaction with SDOH at the district-level in Beijing.

To address these gaps in the research, in this study, we used the global entropy weight method to calculate the composite scores of health outcomes and different dimensions of SDOH; then, we used descriptive methods and spatial autocorrelation analysis to understand the spatial distribution characteristics of the health outcomes. The effects of SDOH (healthy lifestyle, health service, healthy environment, health security, and social and economic factors) on public health revealed using the geodetector technology provide a reference for decision-making aimed at optimizing the promotion strategy of the *Healthy Beijing Initiative*.

## 2 Materials and methods

### 2.1 Indicators and data source

**Figure 1:**
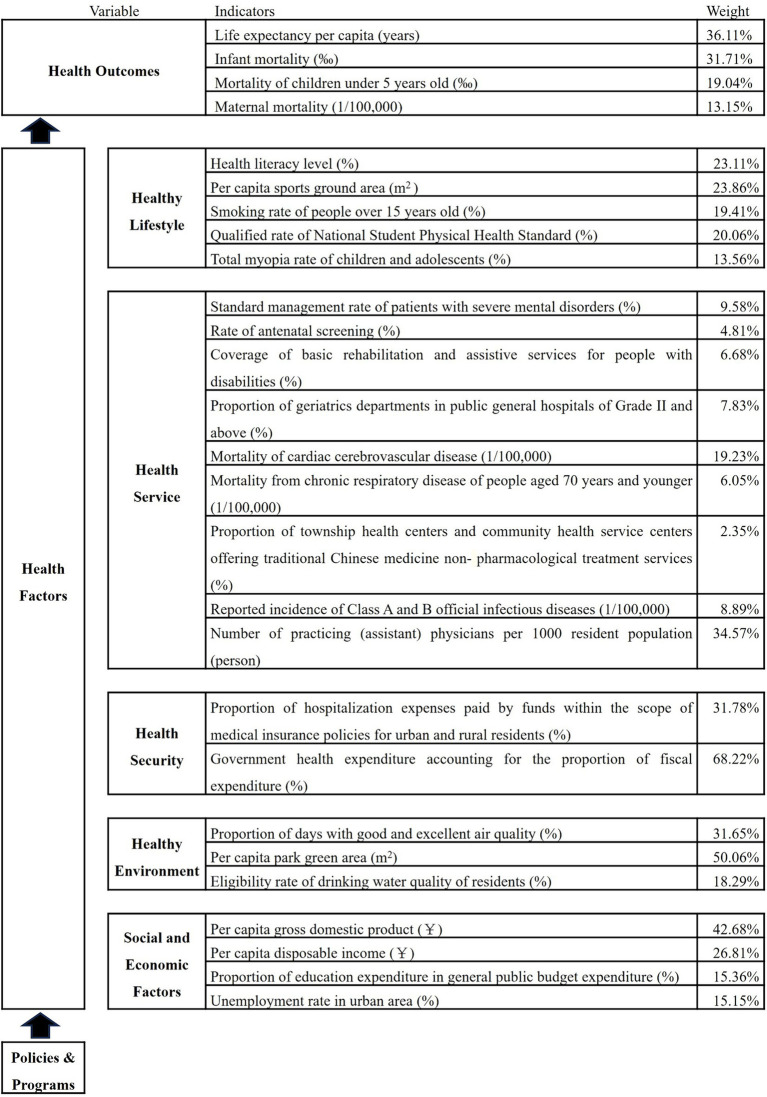
Health outcomes and the SDOH evaluation system.

Based on the ecological model of SDOH established by Dahlgren and Whitehead, we selected indicators from the *Healthy Beijing Initiative (2020–2030)* monitoring indicators pool, referencing previous literature (19–24) and considering data availability and quality. Based on these, we built a health outcomes evaluation system and SDOH evaluation system (Figure 1). In the SDOH evaluation system, we delineated the underlying modifiable determinants of health and grouped them into five main categories: healthy lifestyle, which included indicators for individual lifestyle and sports venue construction; health service, including health management of key populations, mortality rate of major chronic diseases, and health resources allocation; health security, including medical insurance benefits and government health expenditure; healthy environment, consisting of air and water quality, green area of park; and social and economic factors, including indicators of income, education, and employment. Although considered an important predictor of health, genetics was excluded from the model because this factor is currently largely unmodifiable.

In this study, we focused on 16 districts in Beijing, analyzing the spatial features of residents’ health outcomes and influencing factors. The indicator data were derived from district-level monitoring data of the *Healthy Beijing Initiative*.

### 2.2 Statistical methods

#### 2.2.1 Descriptive methods

The Shapiro–Wilk normality test was used to ascertain the distribution type of each SDOH variable. The median was used to describe non-normal distributions, while the mean (standard deviation, SD) was used for normal distributions. The spatiotemporal distribution of the health outcomes was mapped using ArcGIS 10.8 software.

#### 2.2.2 Global entropy weight method

Health outcomes and SDOH composite scores were measured by the global entropy weight method via the following steps (18, 25, 26):

(1) As the measurement units of indicators related to health outcomes and SDOH were different (for example, percentage versus absolute value), this study standardized the indicators by the following equations.

Positive indicators: ${\left(x_{ij}^{t}\right)}^{\prime }=\frac{x_{ij}^{t}-x_{jmin}}{x_{jmax}-x_{jmin}}\times 0.9+0.1$,

Negative indicators: ${\left(x_{ij}^{t}\right)}^{\prime }=\frac{x_{jmax}-x_{ij}^{t}}{x_{jmax}-x_{jmin}}\times 0.9+0.1$.

In the equation above, $x_{ij}^{t}$ is the value of indicator j in year t of the district i.

(2) The weight $w_{j}$ for each indicator related to health outcomes and SDOH was determined by following the steps below:

The information entropy of the indicator j was calculated according to the concept of information entropy:


$$e_{j}=-K\sum_{t=1}^{T}\sum_{i=1}^{m}f_{ij}^{t}\ln f_{ij}^{t}$$

In the equation above, $K=\frac{1}{\ln mT}$, $f_{ij}^{t}=\frac{{\left(x_{ij}^{t}\right)}^{\prime }}{\sum_{t=1}^{T}\sum_{i=1}^{m}{\left(x_{ij}^{t}\right)}^{\prime }}$.

Finally, the entropy weight of indicator j was calculated as follows:


$$w_{j}=\frac{1-e_{j}}{\sum_{j=1}^{n}\left(1-e_{j}\right)}$$

In the equation above, $1-e_{j}$ represents the coefficient of variation.

(3) The composite scores $S_{i}$ for health outcomes and SDOH were weighted sums of the standardized individual measures, as follows:
  $$S_{i}=\sum_{j=1}^{n}w_{j}{\left(x_{ij}^{t}\right)}^{\prime }$$

#### 2.2.3 Spatial autocorrelation analysis

We adopted the global spatial autocorrelation coefficient (Moran’s I) and Moran scatter plot to analyze the spatial clustering characteristics of health outcomes of residents in Beijing. The formula for global Moran’s I is as follows:


$$I=\frac{\sum_{i=1}^{n}\sum_{j=1}^{n}W_{ij}\left(X_{i}-\bar{X}\right)\left(X_{j}-\bar{X}\right)}{s^{2}\sum_{i=1}^{n}\sum_{j=1}^{n}\left(W_{ij}\right)}$$

In the equation above, $X_{i}$ and $X_{j}$ represent the health outcomes of the i and j district, respectively; $\bar{X\;}$ and $s^{2}$ represent the mean and variance of health outcomes, respectively; n represents the total number of spatial units in the study; and $W_{ij}$ is the spatial weight matrix, denoted by the adjacency weight matrix in this study. Moran’s I ranges from −1 to 1, with values greater than 0 indicating positive spatial autocorrelation, values less than 0 indicating negative spatial correlation, and values equal to 0 indicating no spatial correlation (27).

The local Moran’s I is visualized as a two-dimensional scatter plot, which visually presents the clustering types among the studied objects. The I-IV quadrants of the Moran Scatterplot represent the high-high (H-H) cluster, low-high (L-H) cluster, low-low (L-L) cluster, and high-low (H-L) cluster in turn, where H-H and L-L clusters represent positive spatial autocorrelation, and L-H and H-L clusters represent negative spatial autocorrelation.

#### 2.2.4 Geographical detector

Geographical detector is a new analytical method for detecting spatial stratified heterogeneity and identifying the underlying driving factors. It comprises four types of tools: factor detector, interaction detector, risk detector, and ecological detector. Factor detector and interaction detector can detect the extent to which a factor accounts for the spatial divergence of the dependent variable and the interaction between different factors (28). Therefore, we used factor detector and interaction detector to analyze the influence of SDOH on health outcomes.

(1) Differentiation and factor detector: The spatial stratified heterogeneity of health outcomes of residents of Beijing and the explanatory power of certain factors on its heterogeneity were detected. The q-value was measured, as follows:
  $$q=1-\frac{1}{N\sigma^{2}}\sum_{h=1}^{L}N_{h}\sigma_{h}^{2}$$

In the equation above, *h* = 1,…, L refers to strata of each factor; $\sigma^{2}$ and $\sigma_{h}^{2}$ represent the variance of health outcomes overall and for stratum h, respectively; and N and $N_{h}$ represent their sample size, respectively. The range of q is [0, 1]. If q is closer to 1, this factor has a greater effect on health outcomes.

(2) Interaction detection: The interaction between different factors was identified to assess whether the combined effect of factors X1 and X2 will increase or decrease the explanatory power of health outcomes. The *q*-value(X1∩X2) of two factors X1 and X2 was calculated after interaction and then compared with q(X1) and q(X2) to determine the interaction type. The five interaction types are shown in Table 1.

**Table 1:** Types of interaction between two covariates.

Basis of judgment	Interaction
q(X_1_∩X_2_) < Min(q(X_1_),q(X_2_))	Nonlinear weakening
Min(q(X_1_),q(X_2_)) < q(X_1_∩X_2_) < Max(q(X_1_),q(X_2_))	Single nonlinear weakening
q(X_1_∩X_2_) > Max(q(X_1_),q(X_2_))	Double enhancement
q(X_1_∩X_2_) = q(X_1_) + q(X_2_)	Independence
q(X_1_∩X_2_) > q(X_1_) + q(X_2_)	Nonlinear enhancement

## 3 Results

### 3.1 Spatial distribution of residents’ health outcomes in Beijing

**Figure 2:**
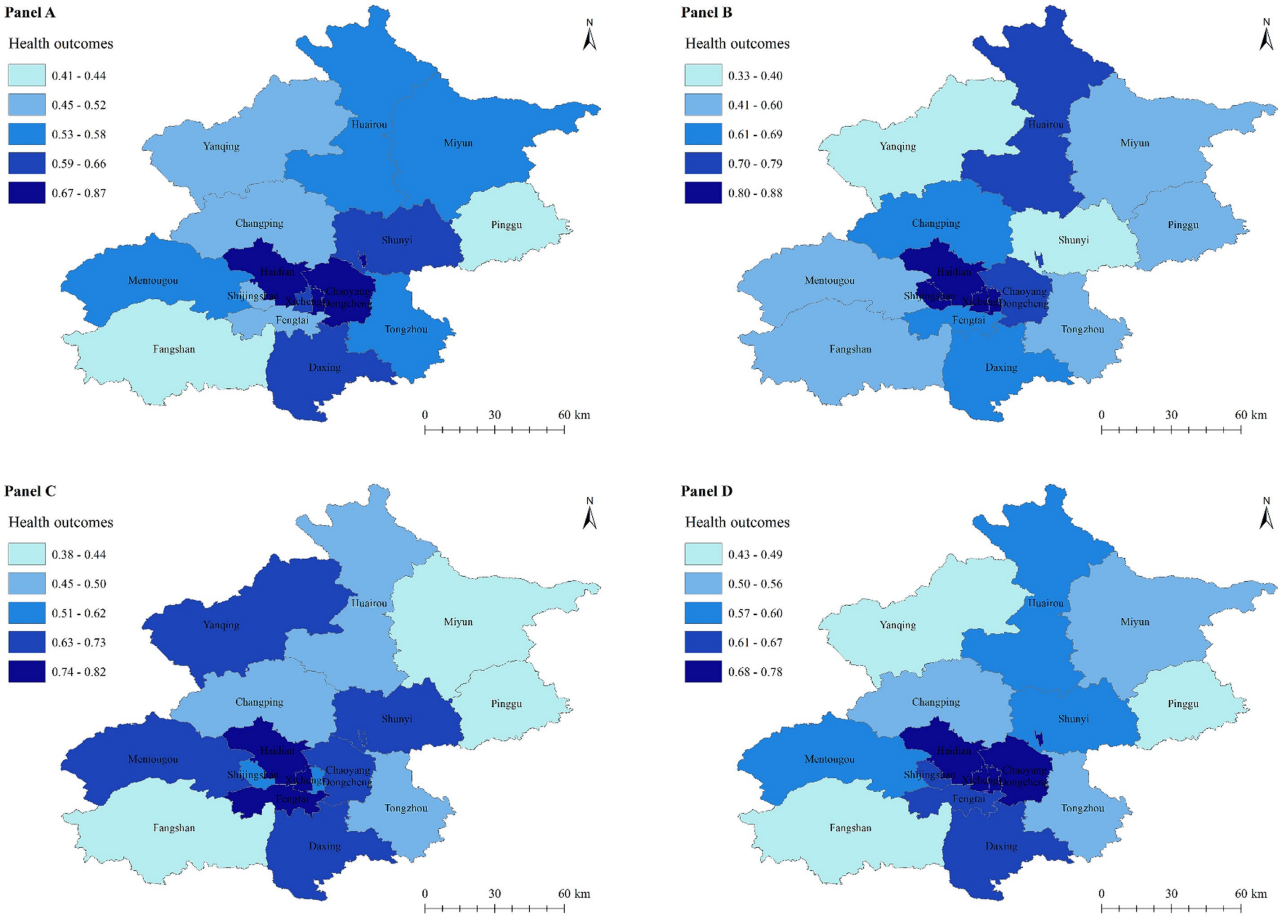
Spatial distribution of health outcomes in Beijing. Panel **(A)**: 2020. Panel **(B)**: 2021. Panel **(C)**: 2022. Panel **(D)**: 2020–2022.

The health outcome values of 16 districts in Beijing were divided into 5 grades: lowest level, lower level, medium level, higher level, and highest level by the Jenks natural breaks method. The spatial distribution of the health outcomes of Beijing in 2020, 2021, 2022, and 2020–2022 was plotted (Figures 2A–D). The 3-year average health outcomes of the 16 districts in Beijing varied significantly (0.43–0.78), with obvious spatial stratification and heterogeneity. The 3-year average health outcomes in central urban areas such as Dongcheng, Xicheng, Haidian, and Chaoyang were at a high level, while those for the outer suburbs of Yanqing, Pinggu, and Fangshan were at a low level.

**Figure 3:**
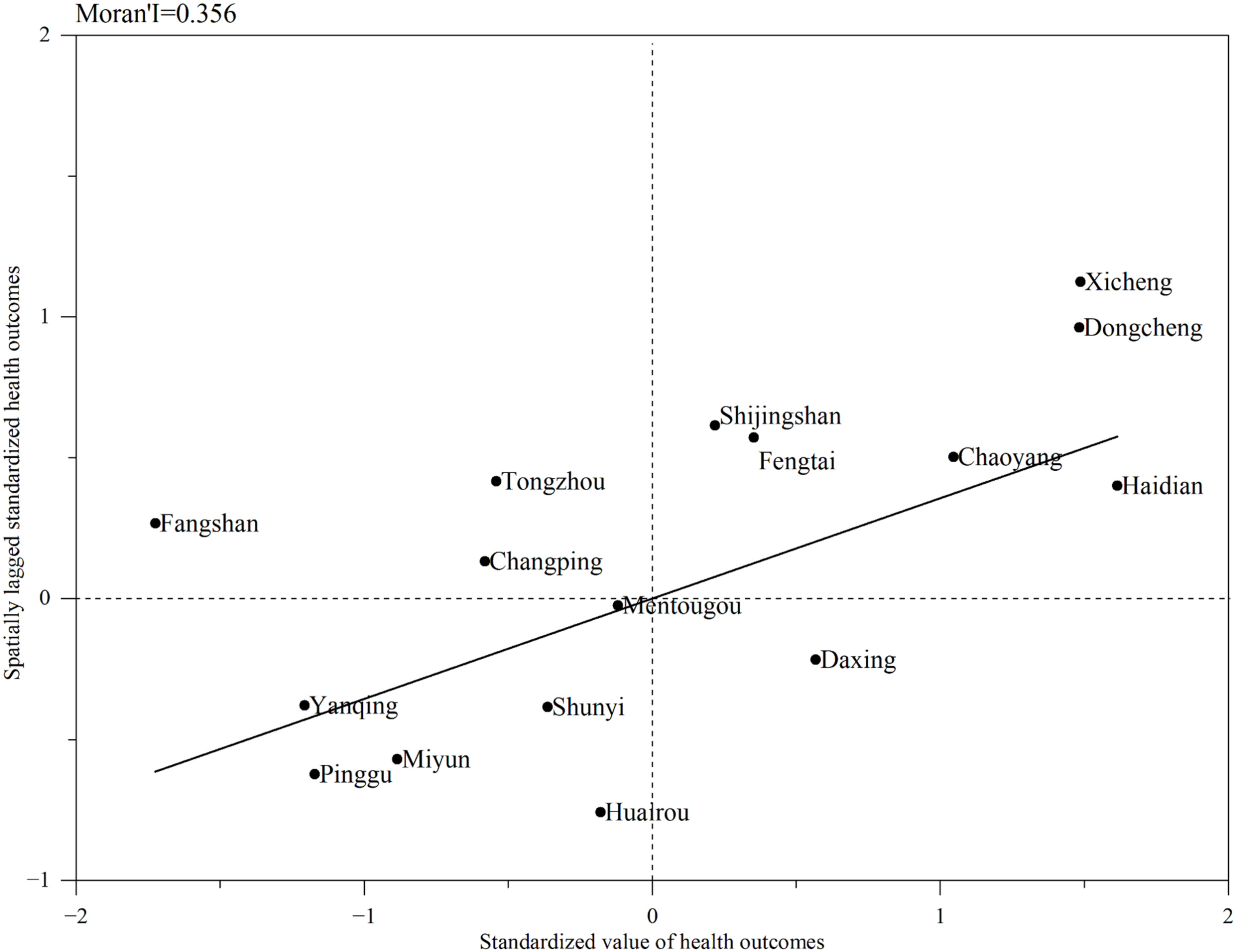
Moran scatterplot of mean health outcomes in Beijing during 2020–2022.

The results of global autocorrelation analysis showed that the global Moran’s I was 0.356 (*p* < 0.05), indicating significant spatial clustering of the 3-year average health outcomes in Beijing. To further explore the clustering types of health outcomes, Moran scatterplot was used to describe and visualize spatial correlation of the 2020–2022 average health outcomes, dividing16 districts into four quadrants by standardized values of health outcomes and spatially lagged standardized health outcomes (Figure 3). The health outcomes comprised two cluster types, H-H and L-L. The H-H clusters were mainly found in central urbans, such as Dongcheng, Xicheng, Haidian, Chaoyang, Fengtai, and Shijingshan; meanwhile, the L-L cluster was largely distributed in ecological conservation areas, such as Pinggu, Yanqing, Miyun, Huairou, and Mentougou.

### 3.2 Spatial distribution of SDOH

Descriptions of SDOH variables for 16 districts are presented by mean [standard deviation (SD)] or median (25th percentile, Q1, 75th percentile, Q3) (Table 2). Spatial differences were observed in the five dimensions of SDOH. Social and economic factors, healthy lifestyle, and health service had the highest values in the capital functional core area, at up to 0.83, 0.57, and 0.80, respectively, while the urban sub-center and flat new town performed poorly, with lowest values of 0.16, 0.31, and 0.38. In terms of health security, Daxing District performed best at 0.77, while Shijingshan District and Tongzhou fared worse. In terms of healthy environment, ecological conservation areas had the best performance—at up to 0.92—while the capital functional core area had the worst performance, at only 0.33.

**Table 2:** Descriptive analysis of SDOH of 16 districts in Beijing, 2020–2022 [Mean (SD)/Median (Q1, Q3)].

SDOH	2020	2021	2022	2020–2022 Average
Healthy lifestyle	0.44 (0.10)	0.51 (0.48, 0.56)	0.55 (0.07)	0.49 (0.08)
Health service	0.60 (0.10)	0.60 (0.09)	0.52 (0.12)	0.58 (0.10)
Health security	0.44 (0.13)	0.44 (0.12)	0.63 (0.15)	0.50 (0.11)
Healthy environment	0.49 (0.16)	0.54 (0.48, 0.62)	0.56 (0.12)	0.52 (0.46, 0.62)
Social and economic factors	0.30 (0.22, 0.51)	0.31 (0.24, 0.53)	0.31 (0.24, 0.53)	0.30 (0.23, 0.5)

### 3.3 Effect of SDOH on health outcomes based on factor detector

Based on the factor detector, we analyzed the effect of SDOH on the spatial divergence of health outcomes. Table 3 listed the q-value and *p*-value for each dimension. In terms of the 3-year average, the influence of SDOH on the spatial differentiation of health outcomes was as follows: Social and economic factors (0.85) > healthy lifestyle (0.68) > health service (0.53) > healthy environment (0.23) > health security (0.10). There was no statistically significant difference in *q*-value of health security and healthy environment (*p* > 0.05). The heterogeneity of health outcomes could mainly be attributed to social and economic factors, healthy lifestyle, and health service. The time trend indicated that the influence of health service and health security on residents’ health outcomes gradually decreased, while social and economic factors, healthy lifestyle, and healthy environment gradually emerged as the main factors influencing health outcomes.

**Table 3:** Analysis of the effect of SDOH on health outcomes based on factor detector.

Year/q	Healthy lifestyle	Health service	Health security	Healthy environment	Social and economic factors
2020–2022	0.68^**^	0.53^*^	0.10	0.23	0.85^**^
2020	0.17	0.33^**^	0.38^*^	0.33	0.26^*^
2021	0.23	0.49^*^	0.14	0.30^*^	0.62^*^
2022	0.64^**^	0.13	0.04	0.23^*^	0.63^**^

^**^, ^*^ indicates significance at the 5 and 10% levels, respectively.

### 3.4 Interactions between different factors based on interaction detector

**Figure 4:**
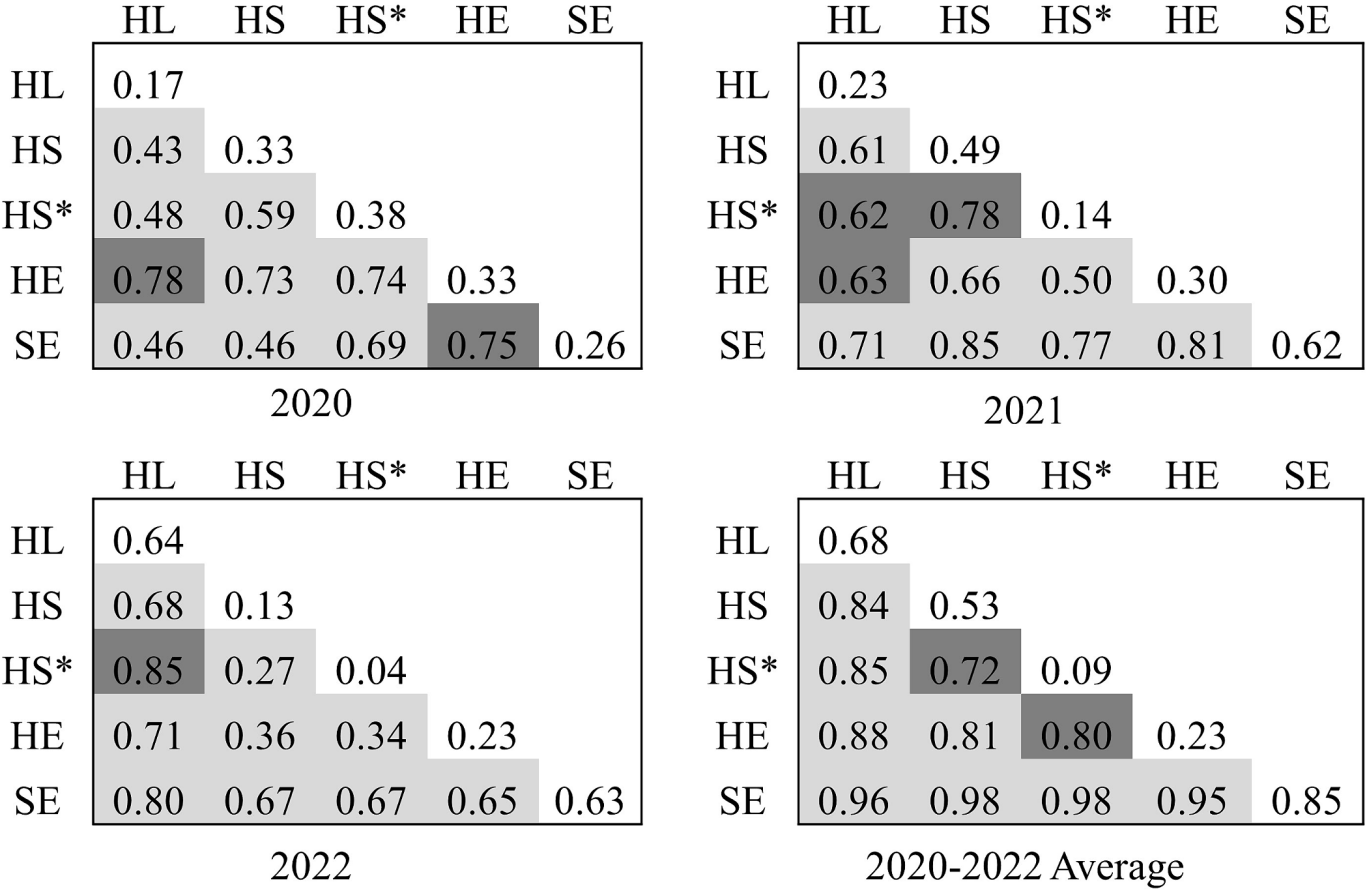
Interaction detector analysis results. Light gray: double enhancement; deep gray: nonlinear enhancement. HL, Healthy lifestyle; HS, Health service; HS*, Health security; HE, Healthy environment; SE, Social and economic factors.

Interaction detector was used to identify the effects and types of interactions between factors. As shown in Figure 4, the interaction between any two factors of SDOH is described as “double enhancement” or “nonlinear enhancement,” and the interaction effect of any two factors was greater than their respective influence on the health outcomes. Even for the factors with lower *q*-values, there was an increase in their *q*-value after the interaction. At the 3-year average level, the top three interaction strengths were social and economic factors∩health service (0.98), social and economic factors∩health security (0.98), and social and economic factors∩healthy lifestyle (0.96), all of which represented the interaction between social and economic factors and other factors. The interaction between healthy lifestyle and other factors was also considerable, e.g., the *q*-value of the interaction with healthy environment was 0.88.

## 4 Discussion

In this study, a system for the evaluation of health outcomes and a multi-dimensional SDOH evaluation system based on the main monitoring indicators of *Healthy Beijing Initiative (2020–2030)* were constructed. The health outcomes composite score at the district level in Beijing from 2020 to 2022 was measured and the overall regional differences and spatial correlation characteristics of residents’ health outcomes were identified. Finally, the effect of SDOH on the spatial differentiation of health outcomes was explored by the geodetector method. To our knowledge, this study represents the first application of geodetector technology to explore the relationship between health outcomes and social determinants in Beijing from a spatial perspective.

The results of the factor detector analysis indicated that the core factors influencing residents’ health outcome were social and economic factors, healthy lifestyle, and health service, followed by healthy environment; meanwhile, the influence of health security was relatively small. Social and economic factors (such as income, education, and employment) could largely explain the spatial stratified heterogeneity of health outcomes at the district level across development periods. Social and economic factors are upstream determinants of health that can influence health both directly [through stressful experiences, for example (29, 30)] and indirectly (by affecting people’s ability to access health care and make health-related choices) (3). Upstream determinants related to living and working conditions influenced health-related behaviors and ability to receive recommended medical care. Hood et al. found that about 40% of modifiable health determinants were due to social and economic factors while 30% due to health behaviors (31), which was consistent with our findings. These findings highlight the importance of prioritizing health-related investments, placing health at the heart of all economies and underlining health as a fundamental human right through legal and economic means.

The other factors that were found to influence residents’ health outcomes were dynamic across different development periods. When health services and health security reached a high level of stability, healthy lifestyle and healthy environment had a stronger influence on health outcomes. In modern society, many health problems are caused by unhealthy lifestyles and behaviors; as Amador et al. showed, regional variations in health status are predominantly influenced by lifestyle rather than genetics (32). Sun et al. found that a low-risk lifestyle (never smoking or quitting not for illness, no excessive alcohol use, being physically active, healthy eating habits, and healthy body shape) was associated with a higher life expectancy compared with individuals not adopting a low-risk lifestyle in Chinese adults (33), healthy lifestyles are key to improving life expectancy in China (34). Promoting a healthy lifestyle is therefore the central factor in enhancing the health of the population. Health inequalities can be addressed through appropriate social and economic interventions; in addition, residents hold primary responsibility for their own health, which has important implications for the development of healthcare policy. Many scholars also agree that the equity of health service is a crucial factor affecting health equity (35–38), and that health service is an important force in promoting residents’ health. Furthermore, the fair and reasonable allocation of health resources is crucial for ensuring equitable health care services. In this study, the number of practicing (assistant) physicians per 1,000 residents had the largest weight in the health service dimension, which suggested that this indicator was unevenly distributed among districts based on the weight calculation principle of the entropy weight method. Therefore, more detailed health resource planning and allocation policies for specific regions (L-L and L-H health outcomes cluster regions) are necessary to balance the distribution of healthcare-related human resources at the municipal level. Yang and Sun (27) also found that the influence of the health service declined over time. Since the new healthcare reform, Beijing has gradually increased government health investment, improved the basic medical insurance system and compensation policies, and also enhanced the health financing structure. In 2022, the proportion of personal health expenditure among the total health expenditure dropped to 13.56% and health security has reached the same level as that in developed countries, entering the stage of steady advancement. Consequently, the impact of health security on residents’ health was relatively small. The impact of healthy environment on residents’ health outcomes is characterized by persistence and slowness, and it may take some time to become apparent. Therefore, the impact of this factor on residents’ health outcomes was relatively weak during the sample period; however, healthy environment has gradually become an important influencing factor on health outcomes. Pu et al. (39) also confirmed that the nonlinear effect of air quality on life expectancy is gradually increasing. The governments of various districts in Beijing, especially those in the central urbans, should focus on protecting the ecological environment and improving air quality alongside developing the social economy.

Using the interaction detector, we found that the *q*-value of social and economic factors interacted with any other factors was ≥0.95, followed by the interactions of healthy lifestyle with other factors, all of which were ≥ 0.84. These results indicate that the combination of social and economic factors and healthy lifestyle with other factors can significantly affect health. Therefore, the government should focus on promoting social and economic development and healthy lifestyles—specifically, promoting economic development, increasing investment in education and job security, strengthening health education, striving to improve residents’ health literacy, building parks, sports venues and other supporting environments, as well as encouraging residents to adopt healthy lifestyles such as quitting smoking and limiting alcohol consumption. Furthermore, the interaction of any two dimensions of SDOH on health outcomes was found to be stronger than that of any single factor. Accordingly, the interaction of health service, health security, healthy environment, and healthy lifestyle should be fully utilized to advocate a hybrid management model to enhance its “1 + 1 > 2” function of promoting residents’ health outcomes.

Our study reveals the impact of SDOH on the spatial hierarchical heterogeneity of health outcomes and their interactions in Beijing, which has important implications for improving the Healthy Beijing strategy and related health policies. In addition, the selected variables were extensive and covered multiple dimensions, and the data were derived from the Healthy Beijing Surveillance data, which are accurate and authoritative. Despite these strengths, our study still has some limitations. First, the relationship between the dependent and independent variables was statistical and did not provide sufficient evidence for inferring causality. Application of the geographical detector can screen out highly suspicious health influences for further confirmation through longer-term longitudinal studies (40). At the same time, this study does not delve deeply into the causal relationships among these influencing factors. Future studies could consider incorporating causal analysis methods, such as Structural Equation Modeling (SEM) or multilevel models, to further validate the causal relationships among these factors, thereby enhancing the theoretical depth of the research. Second, the geodetector is only capable of exploring interactions between two factors and cannot provide insight into the impact of multiple interactions on health outcomes; this is also a key issue to be addressed in future studies. Third, although this study has included five dimensions of SDOH, the components of SDOH may not fully reflect the differences in health outcomes, and inevitably omit some potential influencing factors, such as population structure, population density, and topography. Finally, the current statistical survey system uses district as the smallest administrative unit for data collection and can only obtain data for district-level indicators at this stage. In the future, with the continued implementation of the Healthy Beijing strategy, data from a larger number of years can be included to increase the sample size and conduct more accurate analysis.

## 5 Conclusion

In conclusion, the health outcomes of Beijing exhibited obvious heterogeneity of spatial stratification, with distinct clustering patterns observed in different areas. In central urban areas, H-H clustering was observed, while in flat new towns and ecological conservation areas L-L clustering was found. Overall, the formation of a high belt low development pattern has not yet been observed. Among the numerous SDOH, social and economic factors, healthy lifestyle and health service largely explain the spatial stratification heterogeneity of health outcomes, representing major factors influencing residents’ health outcomes. However, the impact of health service has weakened over time, while the influence of healthy environment on health outcomes has gradually increased. This highlights the need to focus on or increase the weight of social and economic factors and healthy life in the Healthy Beijing assessment system. In addition, the interaction of the five dimensions of SDOH had a more pronounced influence on residents’ health than each dimension on its own, suggesting that the coordinated development of Healthy Beijing and improvement of the level of co-construction, co-governance, and sharing are critical for enhancing residents’ health outcomes. The results of the study provide a basis for decision-making for the Beijing municipal government to optimize the Healthy Beijing strategy and to formulate policies related to promoting health, allocating health resources, and undertaking environmental protection to solve the problem of regional inequality in health outcomes. For example, focusing on the problem of weak resources in outer suburbans, promoting a balanced regional layout of high-quality medical resources, expanding and sinking them to the grassroots level, and carrying out “Internet+Medical Services” to narrow the gap between urban and rural medical resource allocation and promote the fairness accessibility of high-quality medical services. Focusing on improving residents’ lifestyles and narrowing the gap of health literacy levels between the urban and rural residents. Constructing a multi-level health science popularization system, setting up fixed health propaganda columns in rural areas, regularly updating the contents covering knowledge of common disease prevention, healthy lifestyles, and reasonable diets. At the same time, use new media means to disseminate health knowledge. We should actively guide social organizations, enterprises, and individuals to participate in the construction of healthy Beijing, and form a working mechanism featuring government leadership, departmental coordination, social mobilization, and universal participation.

## Data Availability

The original contributions presented in the study are included in the article/Supplementary material, further inquiries can be directed to the corresponding author.
