# Supplementary material for: Spatial characteristics of health outcomes and geographical detection of its influencing factors in Beijing
Source: Front Public Health. 2024 Oct 16;12:1424801. doi: 10.3389/fpubh.2024.1424801 (PMC11521850; doi:10.3389/fpubh.2024.1424801)
Supplement: Supplementary file 1 [file Data_Sheet_1.docx]

Supplementary Material

# Global entropy weight method

Considering the characteristics of this study, which includes multi-indicator, multi-year and multi-regional mixed panel data, we used the global entropy weight method to calculate the composite scores for health outcomes and each dimension of the SDOH, respectively. The underlying principle of the global entropy weight method is to determine weights based on the degree of dispersion of each indicator data.

(1) Processing of raw data. Assuming that we need to calculate the composite scores of the health outcomes and each dimension of the SDOH for m districts in Beijing across n evaluation indicators over T years. By collecting the actual values of each indicator for each district annually, we can form an initial global evaluation matrix mT×n , denoted as:

$$X=\left( X^{1},X^{2},X^{3},\ldots，X^{T} \right)_{mT\times n}=\left\{ X_{ij}^{t} \right\}_{mT\times n}$$

In the equation above,$x_{ij}^{t}$ is the value of indicator j in year t of the district i.

(2) As the measurement units of indicators related to health outcomes and SDOH were different (for example, percentage versus absolute value), this study standardized the indicators by the following equations.

Positive indicators(Higher values represent higher levels of health outcomes or SDOH):${{（x}_{ij}^{t}）}^{'}=\frac{x_{ij}^{t}-x_{jmin}}{x_{jmax}-x_{jmin}}\times0.9+0.1$,

Negative indicators(Higher values represent lower levels of health outcomes or SDOH):${{（x}_{ij}^{t}）}^{'}=\frac{x_{jmax}{-x}_{ij}^{t}}{x_{jmax}-x_{jmin}}\times0.9+0.1$.

In the equation above, ${{（x}_{ij}^{t}）}^{'}$ is the standardized value of indicator j in year t of the district i, $x_{jmax}$ and $x_{jmin}$represent the maximum and minimum values of indicator j in the m districts over T years, respectively.

(3) Calculating information entropy. Information entropy determines the disorder or randomness of an indicator. The calculation formula for the information entropy of the j indicator is as follows:

$$e_{j}=-K\sum_{t=1}^{T} \sum_{i=1}^{m} f_{ij}^{t}\ln f_{ij}^{t}$$

In the equation above,$K=\frac{1}{\ln mT}$, m=16, T=3, $f_{ij}^{t}=\frac{{{（x}_{ij}^{t}）}^{'}}{\sum_{t=1}^{T} \sum_{i=1}^{m} {{（x}_{ij}^{t}）}^{'}}$.

(4) Calculating the coefficient of variation. The coefficient of variation of the indicator is related to the information entropy. The larger its value, the more information utility value the indicator contains, and the greater its weight in the evaluation system. The specific calculation formula is:

$$d_{j}=1-e_{j}$$

(5)The entropy weight of indicator j was calculated as follows:

$$w_{j}=\frac{1-e_{j}}{\sum_{j=1}^{n} \left( 1-e_{j} \right)}$$

In the equation above, $w_{j}$takes the value range of [0,1]，and $\sum_{i=1}^{n} w_{j}=1$

The composite scores for the health outcomes and SDOH dimensions are shown in Supplementary Table 1 and Supplementary Table 2, respectively.

Supplementary Table 1 Health outcomes of 16 districts(2020-2022)

| District | 2020 | 2021 | 2022 | 2020-2022 |
| --- | --- | --- | --- | --- |
| Xicheng | 0.62 | 0.88 | 0.82 | 0.77 |
| Dongcheng | 0.87 | 0.84 | 0.60 | 0.77 |
| Chaoyang | 0.72 | 0.79 | 0.67 | 0.72 |
| Haidian | 0.71 | 0.82 | 0.82 | 0.78 |
| Fengtai | 0.49 | 0.69 | 0.78 | 0.65 |
| Shijingshan | 0.47 | 0.83 | 0.62 | 0.64 |
| Shunyi | 0.61 | 0.40 | 0.73 | 0.58 |
| Tongzhou | 0.58 | 0.60 | 0.50 | 0.56 |
| Fangshan | 0.41 | 0.51 | 0.38 | 0.43 |
| Daxing | 0.66 | 0.69 | 0.68 | 0.67 |
| Miyun | 0.56 | 0.57 | 0.43 | 0.52 |
| Changping | 0.52 | 0.66 | 0.48 | 0.55 |
| Huairou | 0.55 | 0.77 | 0.47 | 0.60 |
| Yanqing | 0.48 | 0.33 | 0.65 | 0.49 |
| Pinggu | 0.44 | 0.59 | 0.44 | 0.49 |
| Mentougou | 0.57 | 0.58 | 0.66 | 0.60 |

Supplementary Table 2 Composite scores of the 5 SDOH dimensions

|  | Healthy lifestyle | | | |  | Health service | | | |  | Health security | | | |  | Healthy environment | | | |  | Social and economic factors | | | |
| --- | --- | --- | --- | --- | --- | --- | --- | --- | --- | --- | --- | --- | --- | --- | --- | --- | --- | --- | --- | --- | --- | --- | --- | --- |
| District | 2020 | 2021 | 2022 | 2020-2022 |  | 2020 | 2021 | 2022 | 2020-2022 |  | 2020 | 2021 | 2022 | 2020-2022 |  | 2020 | 2021 | 2022 | 2020-2022 |  | 2020 | 2021 | 2022 | 2020-2022 |
| Xicheng | 0.51 | 0.56 | 0.64 | 0.57 |  | 0.76 | 0.72 | 0.63 | 0.71 |  | 0.44 | 0.44 | 0.64 | 0.50 |  | 0.25 | 0.41 | 0.35 | 0.34 |  | 0.81 | 0.83 | 0.86 | 0.83 |
| Dongcheng | 0.47 | 0.60 | 0.65 | 0.57 |  | 0.77 | 0.80 | 0.83 | 0.80 |  | 0.44 | 0.45 | 0.72 | 0.54 |  | 0.31 | 0.35 | 0.45 | 0.37 |  | 0.80 | 0.85 | 0.80 | 0.82 |
| Chaoyang | 0.61 | 0.61 | 0.62 | 0.61 |  | 0.62 | 0.61 | 0.48 | 0.57 |  | 0.44 | 0.44 | 0.49 | 0.46 |  | 0.51 | 0.54 | 0.56 | 0.54 |  | 0.53 | 0.48 | 0.54 | 0.52 |
| Haidian | 0.49 | 0.51 | 0.66 | 0.55 |  | 0.64 | 0.66 | 0.55 | 0.62 |  | 0.37 | 0.45 | 0.52 | 0.45 |  | 0.55 | 0.53 | 0.55 | 0.54 |  | 0.67 | 0.71 | 0.71 | 0.70 |
| Fengtai | 0.42 | 0.52 | 0.58 | 0.50 |  | 0.52 | 0.55 | 0.58 | 0.55 |  | 0.33 | 0.43 | 0.91 | 0.56 |  | 0.40 | 0.50 | 0.52 | 0.48 |  | 0.32 | 0.37 | 0.35 | 0.35 |
| Shijingshan | 0.53 | 0.53 | 0.56 | 0.54 |  | 0.64 | 0.64 | 0.61 | 0.63 |  | 0.26 | 0.25 | 0.54 | 0.35 |  | 0.57 | 0.61 | 0.67 | 0.61 |  | 0.46 | 0.51 | 0.49 | 0.49 |
| Shunyi | 0.38 | 0.51 | 0.50 | 0.46 |  | 0.63 | 0.63 | 0.61 | 0.63 |  | 0.53 | 0.39 | 0.61 | 0.51 |  | 0.56 | 0.64 | 0.66 | 0.62 |  | 0.31 | 0.26 | 0.32 | 0.30 |
| Tongzhou | 0.23 | 0.20 | 0.50 | 0.31 |  | 0.37 | 0.41 | 0.37 | 0.38 |  | 0.33 | 0.30 | 0.48 | 0.37 |  | 0.39 | 0.47 | 0.48 | 0.45 |  | 0.19 | 0.19 | 0.21 | 0.20 |
| Fangshan | 0.35 | 0.36 | 0.45 | 0.39 |  | 0.50 | 0.54 | 0.35 | 0.46 |  | 0.33 | 0.41 | 0.74 | 0.49 |  | 0.37 | 0.48 | 0.52 | 0.46 |  | 0.28 | 0.29 | 0.30 | 0.29 |
| Daxing | 0.49 | 0.51 | 0.52 | 0.51 |  | 0.63 | 0.64 | 0.49 | 0.59 |  | 0.67 | 0.73 | 0.92 | 0.77 |  | 0.38 | 0.51 | 0.49 | 0.46 |  | 0.26 | 0.28 | 0.28 | 0.27 |
| Miyun | 0.41 | 0.48 | 0.46 | 0.45 |  | 0.55 | 0.54 | 0.52 | 0.54 |  | 0.47 | 0.44 | 0.62 | 0.51 |  | 0.52 | 0.63 | 0.48 | 0.54 |  | 0.14 | 0.19 | 0.19 | 0.18 |
| Changping | 0.50 | 0.55 | 0.51 | 0.52 |  | 0.62 | 0.61 | 0.52 | 0.58 |  | 0.43 | 0.44 | 0.82 | 0.56 |  | 0.41 | 0.47 | 0.49 | 0.46 |  | 0.32 | 0.32 | 0.30 | 0.31 |
| Huairou | 0.34 | 0.49 | 0.52 | 0.45 |  | 0.64 | 0.63 | 0.54 | 0.61 |  | 0.59 | 0.63 | 0.62 | 0.61 |  | 0.60 | 0.63 | 0.68 | 0.64 |  | 0.23 | 0.23 | 0.28 | 0.25 |
| Yanqing | 0.43 | 0.53 | 0.52 | 0.49 |  | 0.51 | 0.55 | 0.39 | 0.48 |  | 0.56 | 0.55 | 0.40 | 0.50 |  | 0.93 | 1.00 | 0.84 | 0.92 |  | 0.13 | 0.17 | 0.18 | 0.16 |
| Pinggu | 0.56 | 0.59 | 0.49 | 0.55 |  | 0.52 | 0.52 | 0.43 | 0.49 |  | 0.59 | 0.46 | 0.53 | 0.53 |  | 0.46 | 0.56 | 0.48 | 0.50 |  | 0.21 | 0.23 | 0.23 | 0.22 |
| Mentougou | 0.36 | 0.31 | 0.57 | 0.41 |  | 0.64 | 0.61 | 0.46 | 0.57 |  | 0.21 | 0.25 | 0.53 | 0.33 |  | 0.63 | 0.62 | 0.69 | 0.64 |  | 0.26 | 0.32 | 0.35 | 0.31 |

# Discretization results of SDOH

Geographical detector analysis generally requires the independent variables to be categorical, so the five dimensions of SDOH need to be discretized before the analysis. Different discretization schemes will have different impacts on performance. Generally, in the geographical detector analysis, the discretization scheme with the largest q value is the preferred scheme (1). This study also considers statistical differences. We used the quantile method and K-means method to classify the above five dimensions of SDOH, and the classification intervals are shown in Supplementary Table 3.

Supplementary Table 3 Discretization results of SDOH(Classification Interval)

|  | 2020 | 2021 | 2022 | 2020-2022 |
| --- | --- | --- | --- | --- |
| Healthy lifestyle | 2 | 3 | 4 | 4 |
| Health service | 2 | 3 | 2 | 3 |
| Health security | 3 | 3 | 3 | 2 |
| Healthy environment | 4 | 2 | 2 | 4 |
| Social and economic factors | 2 | 5 | 4 | 6 |

References

1 Cao F, Ge Y, Wang J. Optimal discretization for geographical detectors-based risk assessment. Mapping Sciences & Remote Sensing.(2013) 50:78-92. DOI:10.1080/15481603.2013.778562

**
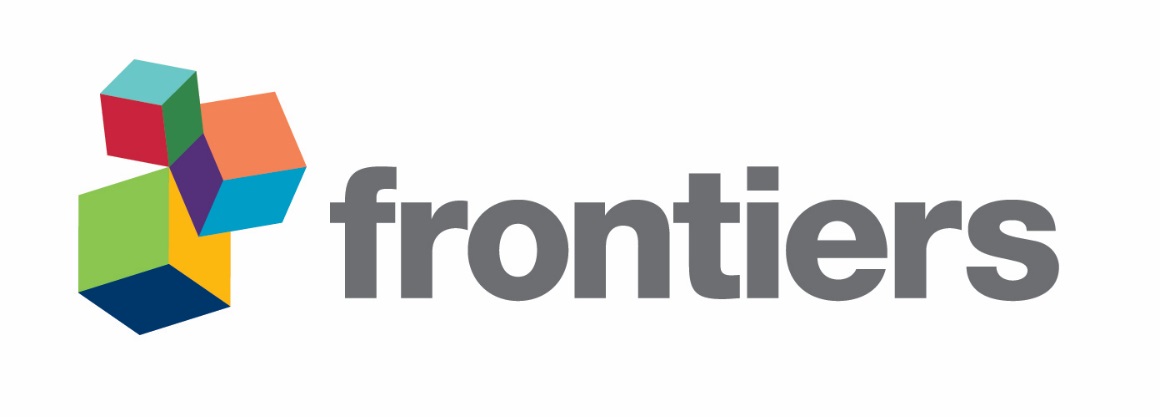
**
